# Magnitude of stunting and its determinant factors among children age 6-59 months at Debre Tabor comprehensive specialized hospital, South Gondar zone, North central Ethiopia, 2020

**DOI:** 10.4314/ahs.v23i4.55

**Published:** 2023-12

**Authors:** Dejen Getaneh Feleke, Ermias Sisay Chanie, Gashaw Mehiret Wubet, Abrham Tsedalu Amare, Agimasie Tigabu Demelash, Aragaw Tesfaw Desale, Reta Dewau Yimer, Ayechew Ademas Tesema

**Affiliations:** 1 Department of Pediatrics and Child Health nursing, college of Health Sciences Debre Tabor University, P.O. Box 272, Debre Tabor, Ethiopia; 2 Department of Medicine, College of Health Sciences, Debre Tabor University, P.O.Box 272, Debre Tabor, Ethiopia; 3 Department of comprehensive nursing, College of Health Sciences, Debre Tabor University, P.O.Box 272, Debre Tabor, Ethiopia; 4 Department of Epidemiology, College of Health Sciences, Debre Tabor University, P.O.Box 272, Debre Tabor, Ethiopia; 5 Department of Epidemiology, College of Health Sciences Wollo University, P.O.Box 272, Wollo, Ethiopia; 6 Department of Environmental, College of Health Sciences Wollo University, P.O.Box 272, Wollo, Ethiopia

**Keywords:** stunting, under-five children, associated factor, north central Ethiopia

## Abstract

**Introduction:**

malnutrition continues to be a significant public health and development concern not only in the developing country but also in the world. It is a serious problem because it is causing the deaths of 3.5 million children under 5 years old per- year.

**Methods:**

Institution based cross-sectional study design was employed using sample of 342 children selected through systematic simple random sampling technique from May 1st -July30 /2020. Bivariate and multivariate logistic regression analysis was used. The variables which had significant association were identified on the bases of P value<0.05 and AOR 95% CI.

**Result:**

The analysis this study revealed that, 42.6% of children were stunted. The main associated factors of stunting were found to be birth order of the child, maternal occupation, frequency meal per day, mother who did not wash their hand before breastfeeding, (AOR=1.636:95%CI:1.00-2.674), children who were not vitamin A, supplemented (AOR=1.901, 95%CI: 1.162-3.109), and child whose mother were not use family planning (AOR=2.916, 95%CI: 1.064-7.989 were associated with outcome variable.

**Conclusion and recommendation:**

From the findings of this study, it is concluded that stunting is still an important problem among children aged 6-59 months. Especial attention should be given on intervention of malnutrition.

## Background

Nutritional status is the result of complex interactions between food consumption and the overall status of health and health care practices 1. Child stunting, or low height-for-age, is an indicator of chronic malnutrition that is still highly prevalent in many regions around the world 1. The consequences of stunting include poor health and school performance, impaired physical and mental development, and perpetuation of the cycle of poverty, as it may result in deficits in productivity in adulthood 2. Stunting (deficit in height for age of at least -2 Z score) affects close to 195 million children under five years of age in the developing world 3.

Under nutrition in under-five children is measured by weight, height and age of the child, and it can be indicated through three forms; stunting, underweight and wasting. According to the WHO growth standard, stunting is the percentage of under-five children whose height for age is below Minus two standard deviations compared to standard population of under-five children 1, 4.

Stunting (low height-for-age) is a sign of chronic under nutrition that reflects failure to receive adequate nutrition over a long period and Height-for-age is a measure of linear growth retardation and cumulative growth deficits. Stunting can also be affected by recurrent and chronic illness 1. The period from birth to two years of age is particularly important because of the rapid growth and brain development that occurs during this time 2.

Malnutrition remains one of the most common causes of morbidity and mortality among children throughout the world. It has been responsible, directly or indirectly, for 60% of the 10.9 million deaths annually among children under five. Over two-thirds of these deaths, which are often associated with inappropriate feeding practices, occur during the first year of life 5. Malnutrition is one of the leading causes of morbidity and mortality in children under the age of five in developing countries 6. Despite the economic growth observed in developing countries, malnutrition particularly stunting is still highly prevalent in Ethiopia an important public health problem; stunting is 38%, in Amhara National Region State (ANRS) 46% 1. In our country Ethiopia, due to cultural factors, foods that are good sources of energy and protein are not allowed to be consumed by pregnant women for reasons such as difficult and prolonged labor due to fears of a large baby. Similarly, sources of vitamins and minerals are restricted during pregnancy mainly due to the fear of offensive discharges during delivery and skin diseases on the body in different parts of Ethiopian community, even though good practices also there 4.

The prevalence of stunting has decreased considerably from 58% in 2000 to 38% in 2016, an average decline of more than 1 percentage point per year. Stunting for children under age 5 sharply increases between age 6 and 23 months, and peaks at age 24-35 months; this represents the impact of under nutrition in the first 1,000 days of life 1.

Restricting certain food items especially for pregnant and lactating women and children may cause varieties of health effects such as under-nutrition of the pregnant mother leading to increased risks in pregnancy and labor, such as anemia and other micro-nutrient deficiency illnesses, low resistance to infection. Restricting children from important dietary items also leads to increased risk of infection, protein-energy malnutrition like Kwashiorkor and Marasmus, poor physical and mental growth 4.

In Ethiopia only 7% of children age 6-23 months are fed appropriately, based on the recommended infant and young child feeding (IYCF) practices. In line with nutrition and nutritional related factors, 57% of children age 6-59 months are anemic, with 25% mildly anemic, 29% moderately anemic, and 3% severely anemic, Children in rural areas 58%) are more likely to be anemic than those in urban areas 49% 1.

In fact, Ethiopia is the highest rates of stunting in the world. Contributing factors to under nutrition include limited employment opportunities, poor infrastructure, high population pressure, low education levels, inadequate access to clean water widespread poverty, poor sanitation, and poor access to health services. Without increased efforts to improve the nutritional status of vulnerable groups such as mothers and children under five years old, it is difficult and risks falling of halving stunting and reducing child mortality, however there is limited study conducted in the Zone to identify the stunting and associated factors of 6-59 months aged children 1.

**Figure 1:**
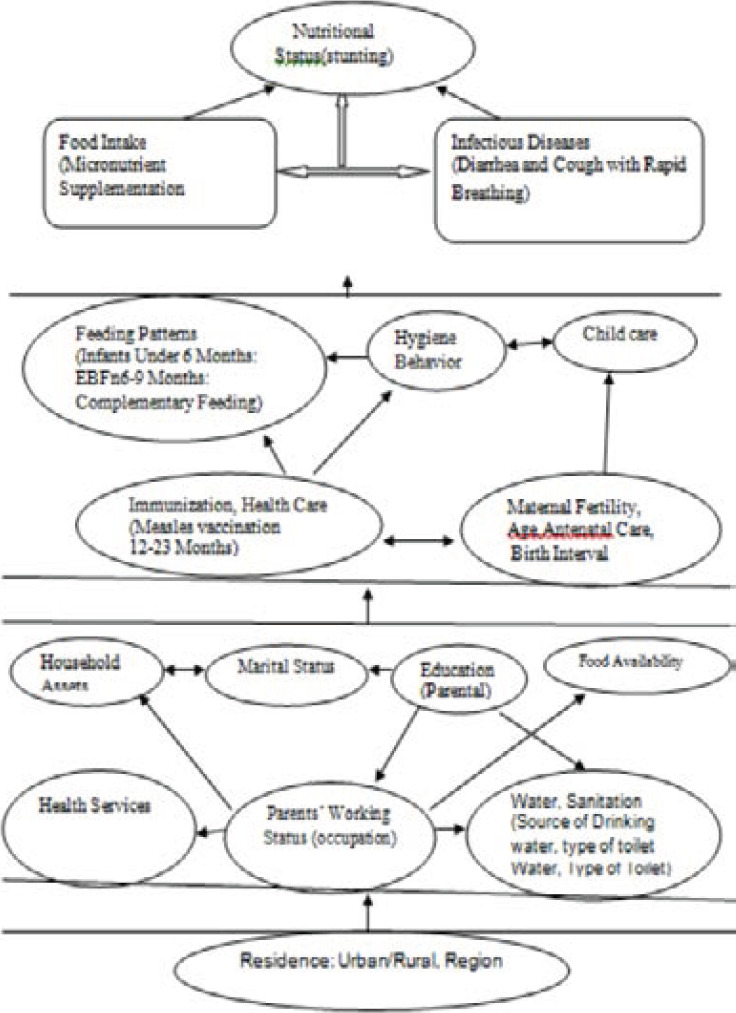
Conceptual frame of factors of malnutrition (stunting) adapted from Different Articles in DTCSH, South Gondar Zone, North central, Ethiopia, [n=342], 2020 1, 4, 6, 10-14

Besides these huge burdens on women & their newborns, there is no enough information available on the stated problem. This study is, therefore, aimed to assessing nutritional status and associated factors Among Children Age 6-59 Months at DTCSH (See Figure-1 Conceptual Framework).

## Methods

### Study area and period

The study was conducted at South Gondar Zone DTCSH. South Gondar Zone is one of the Eleven Zones of the Amhara National Regional State and has a total of Eighteen Woredas. Based on the information from South Gondar Zone Administrative Health Bureau, total population in South Gondar Zone is 2,609,823 among whom are a 49.9% male, and 50.1% are females. Among those <1 year; 6-59 Mon, and 24-59 Mon are 3.4%; 13%, and 8.5% out of the total population respectively. In Guna Begemider Woreda, among eighteen kebele, total population is 104,028. Among whom are < 6 Mon; 6-59 Mon, and 24-59 Mon; 3.1%; 12.9%, and 8.5% out of the total population respectively. Weather condition of south Gondar zone is 4% kola, 78.5% woynadega, 17% dega, and 0.5% wurch. The hospital provides health service to more than 3.5 million populations, currently about 96 health centers and 7 functional district hospitals are available in the catchment area of the referral hospital. About 8136 patients are admitted per year 7. The study was conducted from May 1^st^ -July 30^th^ /2020.

### Study design and participants characteristics

Institution-based cross-sectional study design was conducted. All under five children (paired with their mothers) who visit Debre Tabor Comprehensive Specialized Hospital Source, Population of this study. Selected Children (paired with their mothers) whose ages 6-59 months (paired with their mothers) who visit Debre Tabor Comprehensive Specialized Hospital within the study period were study population. All mother-child pairs aged 6-59 months in DTCSH were included. However, children (paired with their mothers) who were seriously ill, because of difficulty of measurement and the significant effect on child malnutrition were excluded from the study.

### Operational Definition

- **Not severely sick:** children of 6-59 months of age who were not be in coma, intranasal oxygen and the like during the study period.
- **Sick:** children of 6-59 months of age who were diagnosed by the health professional as diseased and on medication or bedridden due to diseases.
- **Colostrum feeding:** Children feed breast without express and discard the first milk from his/her mothers.
- **Complementary feeding:** the child receives both breast milk or a breast milk substitute and solid (semisolid or soft) foods.
- **Income:** It is periodical monthly earning from one's business, lands, work, investment etc.
- **Anthropometry:** Measurement of the variation of physical dimensions and the gross composition of the human body at different age levels and degrees of nutrition by height-for-age 8.
- **Stunting:** (low length-for-age): Moderate and severe; length/height-for-age Z-score between -2SD to -3 SD and <-3 SD, respectively from the median of WHO reference population 9.

### Study variables

#### Dependent variables

Ø Stunting

#### Independent variables

**Socioeconomic and demographic factors:** age of child, sex of child, birth order of the child, preceding birth interval of child, mother /caregiver's religion marital status of the mother/ caregiver's, mother's/ caregiver's education, paternal education status, household monthly income and household family size.

**Child caring practices:** breastfeeding, time for initiation of breastfeeding, frequency of breastfeeding, colostrums' feeding, duration of breastfeeding, age for introduction of complementary food, type of complementary food, and frequency of complementary feeding.

**Environmental factors:** Source of drinking water, Latrine facility and Personal hygiene.

### Sample size determination

A single population proportion sample size determination was carried out through a prevalence value (P) of 46%; the proportion was taken from EDHS 2016 1, ANRS, because there was no study in the area on similar topic precession or marginal error (d) of 5% and 95% of confidence level. The sample size was calculated using the following formula:


$$n=Z_{\frac{\alpha }{2}}{\;}^{2}\frac{P(1-P)}{d^{2}}$$

Where: P=46% (prevalence of stunting, in ANRS) 1, Therefore (p=0.46 and q=0.54)

Level of significance to be 5% (α = 0.05), Z α /2 = 1.96 with 95% CI and, absolute precision or margin of error to be 5% (d = 0.05). The formula for calculating the sampie size is


$$n=\frac{\left(1.96)2^{*}0.46(1-0.54\right)}{(0.05{)}^{2}}$$

n=3.8416 X 0.2116

0.0025

n=325

Finally, 5% for non-response, were added then the final sample size, nf= 325+17=342.

### Sampling techniques and procedures

Among the four-department outpatient service given in DTCSH pediatric department was selected. The sampling procedure was done through the systematic random sampling method.

All patient triage books were labeled (1-N) in the last two weak and 342 cards were selected every two-card using systematic random sampling and the 2nd card was selected by lottery method (the random start and was the first number) then start with #2 and take every 2unit (2, 4, 6, 8…). K^th^ =total number of card / Sample size K = N/n, K=764/342 ∼ 2 where N=764 (N-taken from last 2weak case flow from triage registration book).

### Data collection method and tool

Data was collected using structured questionnaire which is adapted from different literatures 1, 4, 6,, 10-14 in English to enable the comparability of the finding and translated into Amharic language for field work purpose and back to English for checking/ensure/ its consistency. One supervisor and two data collectors (fresh BSC nurses who are unemployed) were participated in the data collection process. Thorough information was given to the data collectors on how to conduct the data collection. Anthropometric measurements (length/height) were done according to WHO standard manual. A portable stadiometer is used to measure older children (above two years) and a calibrated length board was used for younger children (less than two years). Older children were measured at standing position, while younger children less than two years were measured at lie down (supine or recumbent) position. The child was measured without shoes, hats, and hair ornaments. During measurement their head, shoulders, buttocks, and heels will be attached with the vertical surface of the stadiometer by two personnel. The length/height measurement was recorded to the nearest 0.5 cm 15.

### Quality assurance

Data quality was managed by training and appropriate supervision of data collectors. Overall supervision was made by the investigator. Pre-test was done in Adiss Zemen primary hospital by taking 5% (17case) the sample size which was not included in the study area to avoid information contamination.

### Data processing and analysis

The data collected was assessed for properly collected and was checked for completeness, cleaned manually and coded, and then data was entered in EpiData3.1 computer programs to minimize data entry error. The data entered was exported to Statistical Package for Social Sciences (SPSS) version 20 for analysis. The z-score value for Length for Age (LFA) of children generated with WHO child growth standards using WHO Anthro program, version 3.2.2 16. Then recoded, categorized and sorted to facilitate its analysis. Descriptive analysis was used to describe the percentages and number distributions of the respondents by socio-demographic characteristics and other relevant variables in the study. All descriptive statistics were computed in Personal Computer Frequencies and cross tabulations were used to summarize descriptive statistics of the data and tables and graphs were used for data presentation. To determine the factors associated with stunting, binary logistic regressions were applied and the variables (p ≤ 0.2) found to have association with the outcome variable were entered into multivariate analysis. Hosmer-Lemshew goodness of fit test was applied. Finally, the variables which have significant association were identified on the basis of p-values 0.05 and AOR, with 95% CL.

## Results

### Demographic and socio-economic characteristics

A total of 342 children aged 6 - 59 months along with their mothers/care givers were enrolled in the study, with a response rate of 100%. Among 342 under-five children in the study, 194 (56.7%) were male. The mean age of the child and their mother were 28.85 (SD ± 17.35) months and 31.92 (SD±6.41) years respectively. 279 (81.6%) were orthodox followers and 238 (69.6%) were Urban dweller. Regarding marital status of children's mothers/care-givers, 291 (85.1%) were married and 70 (20.8) were private worker. 103 (30.1%) were unable to read and write. Among the total respondent 330 (96.5%) were their mother, 115 (33.6%e) farmer (See Table -1).

**Table 1:** Demographics and Socio-economic characteristics of children among 6 to 59 months in DTCSH, South Gondar Zone, North central, Ethiopia, [n=342], 2020

Explanatory variables		Frequency	Percent
**Sex**	Male	194	56.7
	Female	148	43.3
**Age of the child in months**	6-11	60	17.5
	12-23	91	26.6
	24-35	52	15.2
	36-47	52	15.2
	48.59	87	25.4
**Maternal age in year**	<18	0	0
	18-35	269	78.7
	>=35	73	21.3
**Residence**	Urbane	238	69.6
	Rural	104	30.4
**Religion**	Orthodox	279	81.6
	Muslim	39	11.4
	protestant	24	7.0
	Other***	0	0
**Marital status**	married	291	85.1
	Divorced	36	10.5
	Widowed	8	2.3
	single	7	2.0
**Educational status of the mother**	Unable to read and write	103	30.1
	Able to read and write	36	10.5
	primary	49	14.3
	secondary	79	23
	Certificate and above	75	21.9
**Educational status of the father**	Unable to read and write	68	19.9
	able to read and write	42	12.3
	Primary	47	13.7
	Secondary	60	17.5
	Certificate and above	121	35.4
**occupation**	Housewife	77	22.5
	Employed	79	23.1
	Private	115	33.6
	Other**	71	20.8
**Monthly income (In Birr)**	‹750	38	11.1
	750-1500	51	14.9
	‹1500	253	74
**Family size**	<=5	263	76.9
	>5	79	23.1

** daily labor, farmer…

*** catholic, Adventist

### Child feeding practice and health seeking characteristics

Among the studied children's 122 (35.7%) of them are the first child of the mother, 53 (16.1%) were 4th and above of the mother and 105 (30.7%), 105 (30.7%) were breastfeeding within 30minut and 1hrs of life respectively. Almost all 280 (81.9%) of the children receive first milk (Colostrum) and one third of (33%) of them feed breast 8-10 time per day. Children's 222 (64.9%) were on exclusive breastfeed for the first 6 months and 150 (43.9%) of them start homemade as complementary food, only 63 (18.4%) start liquid diet as complementary. Concerning the content of complementary food 230 (67.3%) adds protein containing items (milk and milk product, egg and/or meat) and 272 (50.3%) of them add fruit and vegetables during complimentary food preparation and 231(67.5%) of them feed their child 4-6 times per day. About 208 (60.8%) of child were fully immunized, and only 93 (27.2%) vitamin A supplemented. Concerning the child illness, 124(36.3%) and 123 (36%) of the child had diarrhea and acute respiratory infection in the last two weeks respectively (See Table-2).

**Table 2:** child feeding practice and health seeking characteristics of children among 6 to 59 months in DTCSH, South Gondar Zone, North central, Ethiopia 2020 [n=342], 2020

Explanatory variable		Frequency	Percent
Birth order	First	122	35.7
	Second	99	28.9
	Third	66	19.3
	fort hand above	55	16.9
Initiation of breast feeding	within30minute	105	30.7
	within1hr	105	30.7
	within2hr	107	31.3
			
	within24hr	20	5.8
	Other	5	1.5
Feeding colostrum's	Yes	280	81.4
	No	62	18.4
EBF	<4momth	79	23.1
	4-6month	222	64.9
	>=6month	41	12
Type of complementary feeding	fluid	63	18.4
	Forage	126	36.8
	Injera	3	0.9
	homemade	62	18.1
Got milk, egg and/or meat during complimentary feeding	Yes	230	67.3
	No	111	32.5
Got fruit and vegetables during complimentary feeding	Yes	172	50.3
	No	170	49.7
Frequency of meal per day	1-3times	81	23.7
	4-6times	231	7.5
	>6times	30	8.8
Immunization statues	Fully immunized	208	60.8
	Not fully immunized	83	24.3
	up-to-date	46	13.5
	not vaccinated	5	1.5
Vitamin A supplementation	Yes	81	23.6
	No	261	76.1
Diarrheal disease	Yes	124	36.5
	No	218	63.7
Respiratory tract infection	Yes	123	36
	No	219	64

### Environmental health conditions of children

Pipe water was the source of water for drinking in the study population with a frequency of 237(69.3%), 183 (53.5%) to wash their hands with soap after toilet, and 94 (27.5%) before breastfeeding and landfill was the major method of solid waste disposal which account 153 (44.7%) and majority has latrine 307 (89.2%) and all the latrines are functional except the two (See Table-3).

**Table 3:** Environmental health condition of children among 6-59month in DTCSH, South Gondar Zone, North central, Ethiopia [n=342], 2020

Explanatory variables		Frequency	Percent
Source of drinking water	Pipe water	237	69.3
	Protected hole water	41	12
	Unprotected hole water	5	1.5
	Spring	54	15.8
	Other	4	1.2
Washing and with soap	Yes	183	53.5
	No	159	46.5
Hand washing before breast feeding	Yes	94	27.5
	No	248	72.6
Solid waste disposal system	Open field disposal	153	44.7
	Land fills	62	18.1
	Composting	1	.3
	Burn	51	14.9
	Other	75	21.9
Availability of toilet	Yes	307	89.8
	No	35	10.2
Utilization	Yes	305	89.2
	No	23	6.7

### Maternal health

Concerning to family planning 329 (96.2%) of mother had had infor mation about family planning of these 242 (78.8%) uses family planning and among FP users 142 (41.5%) use more than two years. ANC follow up of mother during pregnancy was 307 (89.8%). Among 342 mothers, 240 (70.2%) of them birth two and more times and 153 (44.7%) of those give birth with interval of within 1year (See Table-4).

**Table 4:** Maternal health practice of children among 6-59 month in DTCSH, South Gondar Zone, North central Ethiopia [n=342], 2020

Explanatory variables		Frequency	Percent
Information about family plan	Yes	329	96.2
	No	11	3.2
Type of contraceptive use	Piles	56	16.4
	Injection	242	70.8
	Iplanoal	30	8.8
	Other	14	4.1
Utilization of FP	Yes	194	56.7
	No	147	43
Duration of FP	< = 1year	80	23.4
	1-3year	95	27.8
	>=3year	47	13.7
ANC follow up	Yes	307	89.8
	No	35	10.2
parity	One-time	102	29.8
	2-3times	168	49.1
	>=4times	72	21
Interval of birth	<2years	146	41.2
	2-5years	67	19.6
	>5years	21	6.1

### Magnitude of stunting among 6-59 months children

**Figure 2:**
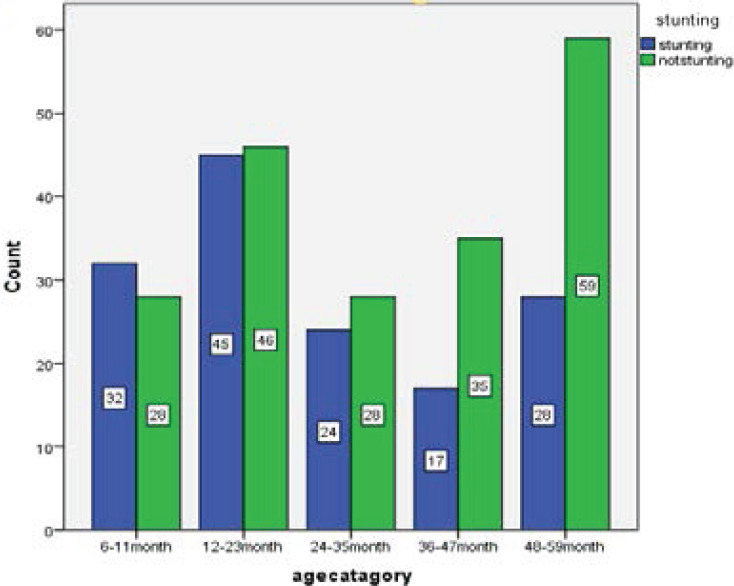
Prevalence of stunting by age among children aged 6-59 months at DTCSH, South Gondar Zone, North central, Ethiopia [n=342], 2020

In the analysis, the prevalence of stunting (low length/height-for-age) among children of 6-59 months According to WHO 16 reference standard taking two standard deviations as cutoff point, the z score of the study subject who fails below minus two standard deviation was taken as stunted, in the study area was 42.6% [95% CI (37.4-47.9) of this 10.5% [95% CI (7.3-13.7)] were moderately stunted and 32.1% [95%CI (27.8-37.1)] were severely stunted. Mean HAZ score was (-1.362 ± 3.62). The sex specific prevalence of stunting in males was 46.9%, while in females it was 37.2%. The age-specific prevalence of stunting in age groups from 6-11 months was 55% and 47.5% in age groups from 12-23month (See Figure-1 and Figure-2).

### Factors associated with stunting among 6-59 months children

In Bi-variable logistic regression analysis age of child, birth order of the child, those who were not got colostrum's feeding, frequency of meal per day, frequency of breastfeeding, immunization status, those hadn't got vitamin-A supplementation, washing hand before breastfeeding, use FP, type of contraceptive used, and duration of contraceptive use were found to be significantly associated with stunting (p<0.2). However, in multivariable analysis only, birth order of the child, occupation of the mother, frequency of the meal per day, handwashing before breastfeeding, vitamin-A supplementation and FP use were significantly associated with stunting (P<=.05). The birth order of the child were associated with stunting, the child who is the second birth order 2times odds likelihood of being stunted followed by third birth order which is 1.6times odds of being stunted but the fourth and above birth order is the lowest likelihood of being stunted when compare with the first birth order [AOR=2.706.95% CI:1.131-6.471] p-value 0.025, [AOR=1.629.95% CI:0.636-4.169] p-value 0.309,and [AOR=0.921.95% CI:0.266-3.186] p-value 0.896 respectively.

Maternal occupation also the other identified associated risk factor children whose mother were government employer 3 times odds likelihood of being stunted followed by private workers which is 2.7 times odds likelihood of being stunted, but children from farmer is low likelihood of stunting when compare to housewife with [AOR=3.204,95%CI:1.219-8.426] p-value 0.018,[AOR=2.875.95% CI:1.085-7.150] p-value 0.035 and [AOR=0.731.95% CI:0.233-2.093] p-value 0.59 respectively. The frequency of child feeding per day was significant predictor to the z score of stunting children who feed 2-3times per day 3times odds likelihood of being stunted followed those feed 4-6times compare to those feed more than 7 times a day with [AOR=3.13.95% CI:0.76-12.09] p-value 0.114 and [AOR=3.282.95% CLT.07-10.06] p-value 0.001 respectively. The children whose mother were not washing hand before breastfeeding were 1.6 times odds likelihood of being stunted when compare with counterpart children (AOR=1.636:95% CI:1.00-2.674), P-value 0.05.

From the analysis, children who were not vitamin-A supplemented 1.9 times higher odds of being stunted when compared to who were supplemented with (AOR=11.91, 95% CI: 1.941-3.595), P value 0.050. Using Family planning is also identified as the factor associated with stunting the child whose mother not use family planning 2.9 time more likelihood of being stunted as compared to whose mother used (AOR=2.916, 95% CI: 1.064-7.989), P-value 0.037 (see Table-5).

**Table 5:** factors associated with prevalence of stunting among children 6-59 months at DTCSH, South Gondar Zone, North central, Ethiopia, 2020

Explanatory variable			Stunting							
			Yes	No	COR (95%CI)	P value	AOR (95%CI)			P value
Age (in month)	6-11	32	(53.3)	28(46.7)	1					
	12-23	45	(49.5)	46(50.5)	1.168(0.608-2.244)	0.641				
	24-35	24	(46.2)	28(53.8)	1.333(0.633-2.808)	0.449				
	36-47	17	(32.7)	35(67.3)	2.353(1.089-5.082)	0.029*				
	48-59	28	(32.2)	59(67.8)	2.408(1.221-4.743)	0.011*				
occupation	Housewife		44(57.1)	33(42.9)	1			1		
	Employed		30(38)	49(62)	1.830(0.452-7.404)	0.397	3.204(1.21-8.42)			0.018*
	private		38(33)	77(67)	8.055(1.619-0.40.079) 0.011*		2.785(1.08-7.15) 0.033*			
	Other*		33(46.5)	37(53.5)	5.831(1.301-26.12)	0.002	0.731(0.23-2.29)			0.591
Birth order	1st		59(48.4)	63(51.6)	1			1		
	2nd		39(39.4)	60(60.6)	1.441(0.842-2.466)	0.183*	2.792(1.43-13.81)			0.029*
	3rd		24(36.4)	42(63.6)	1.639(.0886-3.030	0.115*	1.629(.667-4.98)			0.309
	4^th^and above		24(43.6)	31(56.4)	1.21(0.677-2.295)	0.560	0.921(0.28-3.59)			0.988
Got colostrum's	Yes		122(43.6)	158(56.4)	1			1		
	No		24(38.7)	38(61.3)	0.793(0.344-1.089)	0.095*	1			
Frequency of Breast feeding/ day	2-4times		9(40.9)	13(59.1)	2.11(1.69-6.44.)	0.188		….			
	5-7times		62(38.8)	98(61.2)	2.11(0.98-4.54)	0.058*	….			
	8-10times		50(44.2)	63(55.8)	1.87(0.84-4.173)	0.058*	….			
	>10times		25(53.2)	22(46.8)	1		1			
Frequency of meal Per day	1-3times		30 (37)	51(63)	2.550(1.081-6.017) 0.033			3.132(0.76-12.90)	0.114	
	4-6times		98(42.4)	133(57.6)	2.036(0.93-.42)0.072		3.282(1.07-10.06)			0.001*
	>7times		18(60)	12(40)	1		1			
Vitamin-A supplementation	Yes		27(33.3)	54(66.7)	1			1		
	No		119(45.6)	142(54.4)	1.676(0.994-2.826	0.053	1.91(1.94-3.595)			0.050*
Washing hand before breast feeding	Yes		32(34)	62(66)	1			1		
	No		114(46)	134(54)	1.636(1.00-2.674)	0.05*	1.636(1.00- 2.67),			0.050*.
Use FP	Yes		71(37.1)	122(62.9)	1			1		
	No		74(50.3)	73(49.6)	1.718(1.112-2.654)	0.015*	2.916(1.064-7.989)			0.037*
Duration of FP	<1year		38(47.5)	42(52.5)	2.89(1.331-6.271)	0.007*		…		
	1-3year		59(62.1)	36(37.9)	1.596(0.745-3.473)	0.229*		…	‥	
	>3yaers		34(73)	13(27)	1			1		

AOR =Adjusted Odd Ratio, COR= Crude Odd Ratio, CI= Confidence Interval and *= significant variables.

## Discussions

Nutritional Stunting, which is height for age below that expected on the basis of international growth reference, is a very serious type of malnutrition in that it develops slowly through time before it is evident.

This study intended to assess the prevalence of stunting and associated factors among 6-59 months children. Based on this study, the prevalence of stunting (−2 Z-scores length-forage) was 42.6% [95% CI (37.4-47.9)]. The result of this study revealed that, the prevalence of stunting was 95%CI in line with study conducted in, sub-Saharan Africa,40% and South east Asia, 39%^17^,Vetname,44.3% 10, Kenya,Nirobi,40% 18, Democratic Republic of Congo,43.9% 19, Ethiopian rural area,40% 1, Amhara regional state,46% 1, EDHS 2016 prevalence 38% 1, West Gojjam 43.2% 20, Bulle Hora southern Ethiopia,47.6% 21 ,North shewa Oromia, Hidabu Abote district,47.6% 22, Lalibela Town, Northern Ethiopia,47.3% 6 with Uganda,41.6%^12^. Although present study result showed that the prevalence of stunting children aged 6-59 months higher than a study conducted in, Bangladesh ,34.4% 23, in some developing Africa 36% 24, Ghana, 36% 25 Egypt 13.8% 11, Shine lie Somali destrict,33.4% 13, Haromia,district East Harargie zone,36.07% 14, Mizan Amman town, Bench Maji zone 34.5% 26, in Areka tawon Wolaita zone SNNPR 33.2% 27, Keba Woreda, southern part of Ethiopia,18.7% 28, study conducted in Bure town west Gojjam zone ANRS 24.9% 29 and Addis Ababa 15% (1). However, the prevalence of stunting in the study was also lower than study conducted in, East Africa,48% 8, in Sudan Khartoum 51% 30.

The prevalence of male sex were slightly more likely to be stunted than counterpart female children (46.9 percent and 37.2 percent, respectively) similar to study conducted in south Africa 24, democratic republic of Congo 19.This difference in prevalence of stunting might be due to socio-demographical factors, place of resident, maternal health care, environmental health, food prices, infant and young child feeding practices and child health care.

This study also identified that , the child who is the second birth order 2times odds likelihood of being stunting followed by third birth order which is 1.6 times odds of being stunted but the fourth and above birth order is lowest likelihood of being stunted when compare with the first birth order with [AOR=2.706,95% CI:1.131-6.471] p-value=0.025,[AOR=1.629,95% CI:0.636-4.169] P-value 0.309,and [AOR=0.921,95% CI:0.266-3.186] p-value 0.896 respectively.

Maternal occupation also the other identified associated factor children whose mother were government employer 3times odds likelihood of being stunted followed by private workers which is 2.7 times odds likelihood of being stunted but children from farmer is low likelihood of stunted when compare to housewife with [AOR=3.204,95% CI:1.219-8.426]p-value-0.018,[AOR=2.875,95% CI:1.085-7.150] p-value-0.035 and [AOR=0.731,95% CI:0.233-2.093] p-value-0.59 respectively this might be government employer have no time to care their child the same is true for private worker.

The frequency of child feeding per day was significant predictor to the z score of stunting children who feed 2-3times per day 3times odds likelihood of being stunted followed those feed 4-6times compare to those feed more than 7 times a day with [AOR=3.13.95% CI:0.76-12.09] p-value 0.114 and [AOR=3.282,95%CI:1.07-10.06] p-value 0.001 respectively. This show that appropriate child feeding is the key determinant of child health and growth.

This study also identified that, the handwashing practice of the parent also associated with stunting, the child who was whose parent not washed their hand 1.6 times higher odds of being stunted (AOR=1.636, 95% CI: 1.00-2.674) P-value =0.05 than those who did. This might be due to poor handwashing practice, posing the children at high risk of infectious disease and end up at risk of malnutrition and developmental delay.

From the analysis, children who were not vitamin-A supplemented 1.9 times higher odds' likelihood of being stunted when compared to who were supplemented with (AOR=1.91.95% CI:1.941-3.595) p-value-0.05. This is due to the nutritional and its immunologic value might be deprived in child who was not supplemented and child at higher risk of different infectious and diarrheal disease.

Lastly using Family planning is also identified as the factor associated with stunting the child whose mother not use family planning 2.9 time more like to be stunted as compared to whose mother used (AOR=2.916.95% CI:1.064-7.989) P- value 0.037. This is due to family planning is the single most important determinant of increase birth interval and limit the family size, which are factor associated with stunting. The above socio demographic, and other associated factor difference might be due to poor nutritional status of mothers at pregnancy, inappropriate infant and young child feeding practices including breastfeeding and complementary feeding and other related factors.

## Conclusion

The prevalence of child stunting in this study was found to be relatively high in the study area when compare to study conducted in Bure town and other study done in the region. Birth order of the children, occupational status of the mother, frequency of meal per day, poor handwashing practice, children who were not Vitamin-A supplemented and mother who didn't use family planning remain key associated factors of stunting.

## Recommendations

### For Debre Tabor town health office

Need to increase awareness about family planning and increase use of long-term family planning service for women. Should continuously monitoring and follow up nutritional supply at the community. Need to provide regular, vitamin-A supplementation to all children for eligible age and accordingly.

### For Debr Tabor Comprehensive Specialized hospital

Screen all children for malnutrition at each health care visit and intervene accordingly.

### For health extension workers

Need to give health education for the community about maternal child nutrition to decrease the number of stunted children by focusing age-based feeding practice. Need to provide appropriate counseling on feeding practice of child to the mothers and caregivers with practical demonstration of how to prepare and handling complimentary food for in.

### For the community

Child age-specific attention should be given while feeding infant and young child.

### For researchers

Need to study inter independent variable relationship.

## Figures and Tables

**Figure 3:**
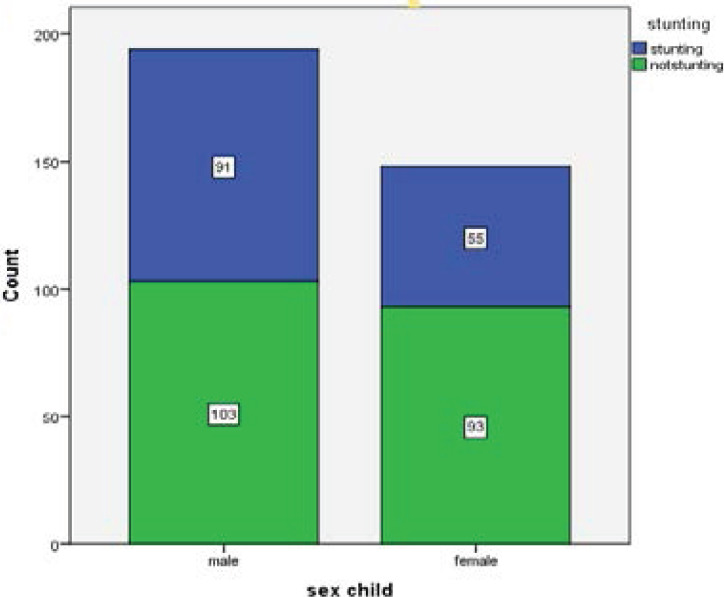
Distribution of stunting among children aged 6-59 months by sex at DTCSH, South Gondar Zone, North central, Ethiopia [n=342], 2020

## Data Availability

Data will be available upon request from the corresponding author.

## Abbreviation

ANRS: Amhara National Regional State
ANC: Antenatal Car
DTU-ERD: Debre Tabor University Ethical Review Board
DTCSH: Debre Tabor Comprehensive Specialized Hospital
EDHS: Ethiopian Demographic Health Survey
EBF: Exclusive Breastfeeding
FP: Family planning
HAZ: Height -For-Age-Z score
IYCF: Infant and Young Child Feeding
MDG: Millennium Development Goal
MPH: Master of Public Health
SAM: Sever Acute Malnutrition
SNNPR: Southern Nations, Nationalities and Peoples Regions
UNICEF: United Nation Children Fund
WHO: World Health Organization

## References

[1] Federal Democratic Republic of Ethiopia, Ethiopia Demographic and Health Survey (2016). key indicators report, central statistical agency, Addis Ababa, Ethiopia, the DHS program ICF Rockville, Maryland, USA.

[2] Bantamen G, Belaynew W, Dube J (2014). Assessment of Factors Associated with Malnutrition among Under Five Years Age Children at Machakel Woreda, Northwest Ethiopia. Journal of Nutrition & Food Science.

[3] World Health organization Global Data Bank on Infant and Young Child Feeding (2010). World Health Statistics.

[4] Ayalew Ermias (2015). the prevalence of stunting and associated factors among children age 6-59 months at mizan-aman town, bench Maji zone, SNNPR region.

[5] Birhanu Mulugeta Molla (2015). Systematic Reviews of Prevalence and Associated Factors of Under Five Malnutrition in Ethiopia: Finding the Evidence. International Journal of Nutrition and Food Sciences.

[6] Yalew Birara Melese (2014). Prevalence of Malnutrition and Associated Factors among Children Age 6-59months at Lalibela Town Administration, North Wollo Zone, Anrs, Northern Ethiopia. Journal of Nutritional Disorders & Therapy.

[7] Fentahun A (2019). South Gondar zone Health Department report.

[8] WHO Multicenter Growth Reference Study Group WHO Child Growth Standards (2006). Length/ height-for-age, weight-for-age, weight-for length, weight-for-height and body mass index-for-age: Methods and development.

[9] Ajao KO, Ojofeitimi EO, Adebayo AA, Fatusi AO, Afolabi OT (2010). Influence of family size, household food security status,and child care practices on the nutritional status of under-five children in Ile-Ife, Nigeria. Afr J Reprod Health.

[10] Mostafa KS (2011). Socio-economic determinants of severe and moderate stunting among under five, children. Banglades.

[11] Sumit M, Bhuiya Abbas (2012). Assessing Vulnerability to Chronic Undernutrition among Under-Five Children in Egypt.

[12] Turyashemererwa F (2009). Prevalence of early child hood malnutrition and influencing factors in peri urban areas of kabarole district, western Uganda. African journal of food agriculture nutrition and development.

[13] Ma'alinetal Abdibari Magnitude and factors associated with malnutrition in children 6-59 months of age in Shinille Woreda, Ethiopian Somalire gional state: a cross-sectional study.

[14] Redi Fuad, Egata Gudina, Kedir Adem (2017). Prevalence of Malnutrition among Children Aged 6-59 in Haramaya District, Oromia, Ethiopia.

[15] (2014). National comprehensive HIV care and treatment training for health care providers, participant manual, ministry of health.

[16] WHO (World Health Organization) (2009). WHO Anthro for personal computers Manual: Software for assessing growth of the world's children and adolescents.

[17] UNICEF (2013). Improving Child Nutrition: The Achievable Imperative for Global Progress.

[18] Woldemariam G, Timotiows G (2002). Determinants of Nutritional Status of Women and Children in Ethiopia.

[19] Kandala NB, Madungu TP, Emina JB, Nzita KP, Cappuccio FP (2011). Malnutrition among children under the age of five in the Democratic Republic of Congo (DRC): does geographic location matter?. BMC Public Health.

[20] Beka T, Wambui K, Zewditu G, Girum T (2009). Magnitude and determinants of stunting in children under five years of age in food surplus region of Ethiopia: The case of West Gojam Zone. Ethiop. J. Health Development.

[21] Asfaw Mandefro, Wondaferash Mekitie, Taha Mohammed, Dube Lamessa (2015). Prevalence of undernutrition and associated factors among children aged between six to fifty-nine months in Bule Hora district. South Ethiopia.

[22] Mengistu Kebede, Alemu Kassahun, Destaw Bikes (2013). Prevalence of Malnutrition and Associated Factors Among Children Aged 6-59 Months at Hidabu Abote District, North Shewa, Oromia Regional State.

[23] Demissie S (2013). “Magnitude and factors associated with malnutrition in children 6-59 months of age in pastoral community of Dollo Ado District, Somali Region, Ethiopia,”. Science Journal of Public Health.

[24] Lesiapeto MS (2010). Risk factors of poor anthropometric status in children under five years of age living in rural districts of the Eastern Cape and KwaZulu-Natal provinces, South Africa.

[25] Olack B, Burke H, Cosmas L, Bamrah S, Dooling K (2011). Nutritional status of under-five children living in an informalurban settlement in Nairobi, Kenya. J Health Popul Nutr.

[26] Ayalew Ermias (2015). the prevalence of stunting and associated factors among children age 6-59 months at mizan-aman town, bench maji zone, SNNPR region.

[27] Desalegn Dereje, Egat Gudina, Halala Yoseph (2016). Prevalence of stunting and associated factors among children aged 6 to 59 months in Areka town, Wolaita Zone, Southern Ethiopia. Journal of Medicine, Physiology and Biophysics.

[28] Eskezyiaw Agedew, Chane Tefera (2015). prevalence of stunting among children aged 6-23 months in kemba woreda, southern ethiopia.

[29] Amare Desalegne, Negesse Ayenew, Tsegaye Baye (2016). Prevalence of Under nutrition and Its Associated Factors among Children below Five Years of Age in Bure Town, West Gojjam Zone, Amhara National Regional State, Northwest Ethiopia. Advances in Public Health, hindawi.

[30] Ahmed E, Mofida Y, Elkhalifa, Maria H, Elnasik H (2011). Nutritional status of the children under age of five in a decertified area of Sudan; alrawakeeb valley, Khartoum, Sudan. International Journal of Current Research.

